# Effect of the Meal Interval Setting of an Automated Concentrate Feeding System on Feed Intake and Feeding Behavior in Fattening Hanwoo Steers

**DOI:** 10.3390/ani14010141

**Published:** 2023-12-31

**Authors:** Hyunjin Cho, Kyewon Kang, Hamin Kang, Seoyoung Jeon, Mingyung Lee, Eunkyu Park, Seokman Hong, Seongwon Seo

**Affiliations:** 1 Division of Animal and Dairy Sciences, Chungnam National University, Daejeon 34134, Republic of Korea; chohyunjin0927@gmail.com (H.C.); kangkyewon26@gmail.com (K.K.); gkals0339@gmail.com (H.K.); seoyoung203@gmail.com (S.J.); mingyung1203@gmail.com (M.L.); 2 Woosung Feed Co., Ltd., Daejeon 34379, Republic of Korea; ekpark@woosung.kr (E.P.); smhong@woosung.kr (S.H.)

**Keywords:** automated concentrate feeding system, meal interval, feeding behavior, Hanwoo

## Abstract

**Simple Summary:**

This study investigated the effect of altering the meal interval setting in an automatic concentrate feeding system on the precision of the system in monitoring the feed intake and feeding behavior of fattening Hanwoo steers. The experiment, involving 29 steers (nine or ten steers per pen), utilized two interval settings: four and six intervals per day. The automatic concentrate feeding system, which divides one day into several intervals, allows cattle to consume a set amount of concentrate mix during each interval. The study’s results indicate that, despite no remarkable change in concentrate intake (*p* > 0.05), the six-interval setting led to a reduction in concentrate residuals of 0.2 kg per visit (*p* < 0.05). However, the six-interval setting resulted in fewer visits for forage consumption and decreased forage and total dry matter intakes (*p* < 0.05). These findings highlight that adjusting these meal intervals affected the feeding behavior and concentrate residuals. This study suggests that a six-interval setting may enhance the precision of the automatic concentrate feeding system in monitoring concentrate intake, although it negatively influences the overall forage consumption.

**Abstract:**

An automatic concentrate feeding system (ACFS) divides the day into several intervals, allowing cattle to consume a predetermined amount of concentrate mix per interval. This study investigated the impact of changing these intervals (four vs. six) in an ACFS on its precision in monitoring the feed intake and feeding behavior of fattening Hanwoo steers. The experiment, involving 29 fattening Hanwoo steers (688 ± 43.3 kg of body weight, 24 months old), employed a switchback design with two interval settings: four and six per day. Both individual forage and concentrate intakes and feeding behaviors were automatically recorded; however, the ACFS measured feed supply, not actual intake. The precision of the ACFS’s intake recordings was tested by manually assessing feed residuals per visit using video recordings. Although no difference was observed in the concentrate intake (*p >* 0.05), the six-interval setting reduced concentrate residuals by 0.2 kg per visit (*p* < 0.05). The increased interval setting also resulted in fewer visits for forage consumption and decreased forage and total dry matter intakes (*p* < 0.05). In conclusion, the increased interval setting for the ACFS reduced the visit frequency for forage consumption and actual forage consumption while improving the precision of the ACFS’s intake recordings.

## 1. Introduction

The adoption of automatic feeding systems (AFSs) in cattle farming has increased globally over the past few decades [1]. Compared to conventional feeding systems, AFSs can significantly reduce the labor hours required for feeding [2] and provide flexibility in feeding times according to an animal’s need [3]. Consequently, the adoption of an AFS may result in an increased feeding frequency, potentially contributing to improved animal health and welfare [4,5].

Various types of AFSs used in dairy and beef cattle farming operate differently depending on the type of feed, including the total mixed ration (TMR), forage, and concentrate mix. Commonly used AFSs for automatic TMR or forage feeding are rail-mounted, stationary, and mobile systems. Although the operating principles may vary for each type, a common feature is the automatic distribution of the TMR or forage to the animal feeding area using conveyor belts, rails, and carts. This approach enables multiple animals to access the TMR or forage simultaneously [6]. In contrast, an AFS dispensing concentrate mix provides feed to each animal individually as they enter the designated station, one at a time [7,8]. This system allows for specifying the amount of concentrate mix provided to each individual per day [9]. Notably, automatic concentrate feeding systems (ACFSs) are commonly integrated into automatic milking systems (AMSs). An ACFS stimulates the voluntary attendance of dairy cows into an AMS by providing a concentrate mix to the dairy cows during milking for supplementing nutrients [10].

Although an ACFS logs individual consumption, the recorded values typically reflect the quantity of feed dispensed rather than the actual amount consumed by the animal. This discrepancy primarily arises because continuous monitoring of the weight of the feed bowl is necessary to determine the actual intake, a process that is significantly more expensive than merely measuring the dispensed feed. Consequently, a variation might exist between the intake recorded by the ACFS and an animal’s actual consumption [11,12]. Many previous studies have determined that the actual intake of the concentrate mix fed through an ACFS can differ significantly from the targeted amount. For instance, Halachmi et al. [11] targeted a daily concentrate mix allowance of 7 kg/d provided through an ACFS integrated with an AMS; however, they reported that the actual intake was approximately 5 kg/d. Similarly, Bach et al. [12] and Henriksen et al. [9] aimed for daily concentrate mix allowances of 3 and 8 kg/d in the AMS. However, Bach et al. [12] found the actual intakes to be 2.6 and 6.8 kg/d, respectively, whereas Henriksen et al. [9] observed intakes of 2.2 and 4.4 kg/d, respectively. The gap between the intended and actual intakes was more pronounced with larger allocation amounts. Bach and Cabrera [13] reported that Holstein cows often consumed less than the allocated concentrate mix when the daily allowance exceeded 4 kg/d. Studies investigating discrepancies between actual and target concentrate mix intakes with an ACFS have primarily focused on dairy cows. Nonetheless, similar outcomes might be anticipated for beef cattle during the fattening period, given their typically higher consumption of concentrate mix than dairy cattle.

An ACFS divides the day into several intervals, during which cattle can consume a limited amount of concentrate mix based on their need. This limit is determined by dividing the daily allowance by the number of intervals. The interval system enables animals to consume the concentrate mix multiple times throughout the day, mitigating issues such as acidosis associated with the consumption of a large quantity of concentrate mix in a single feeding event. These properties of ACFSs can positively impact nutrient digestibility and the ruminal environment by reducing the amount consumed by animals during each feeding [14,15]. Additionally, this approach can contribute to narrowing the gap between actual and target intakes. However, previous studies have primarily focused on this disparity when the target intake varied in the ACFS. To the best of our knowledge, no study has examined the difference between the target and actual intakes, whereby the target intake of the ACFS is held constant while varying the interval setting. Therefore, the objective of this study was to elucidate the effect of changing the meal interval setting of an ACFS on its precision in monitoring the feed intake and forage feeding behavior of Hanwoo fattening steers, especially in environments in which concentrate mix and forage are fed separately. We compared the manufacturer-recommended four-meal interval setting with a six-meal interval setting, whereby each meal had a reduced feeding amount compared to the recommended four-meal interval.

## 2. Materials and Methods

This study was conducted at the Center for Animal Science Research, Chungnam National University, Republic of Korea. The use of animals and the protocols for this experiment were reviewed and pre-approved by the Chungnam National University Animal Research Ethics Committee (CNU-01021).

### 2.1. Animals, Housing, and Diets

Twenty-nine Hanwoo steers (687.5 ± 44.09 kg of body weight (BW)), aged 24 months, were included in this study. The steers were matched based on a similar BW and then randomly allocated into one of three pens, each measuring 10 m × 10 m. Each pen was equipped with one ACFS (Dawoon Co., Incheon, Republic of Korea) and four forage intake monitoring systems (FIMS; Dawoon Co., Incheon, Republic of Korea) that automatically measured individual feed intake. During the experiment, two pens were fed a commercial concentrate mix, while one pen received a high-starch concentrate mix. All three pens were fed the same forage diet. The forage was fed ad libitum twice a day at 08:00 and 18:00 h. The concentrate mix was delivered through the ACFS, allowing for up to 9.2 kg dry matter (DM) per day per head. The nutrient composition and the amount of concentrate mix to be provided were determined according to the Korean feeding standards for Hanwoo steers [16], targeting an average daily gain of 0.7 kg/d. The composition of the concentrate mix and the chemical composition of the experimental diets are described in Table 1 and Table 2, respectively.

### 2.2. Intake Monitoring Systems

The FIMS used in this study was equipped with two load cells to measure the remaining feed, presence sensors to detect steers, and a panel that recognizes the radio frequency identification (RFID) neck tag of the steer. When a steer’s RFID neck tag was not recognized, the panel was raised, and when the RFID neck tag was recognized on the panel, it was lowered to allow the steer to enter. The entry time, staying time, exit time, and intake were recorded only when the steer’s RFID neck tag was recognized and the steer consumed feed. The forage intake was recorded as the difference in weight before entry and after exit.

**Figure 1:**
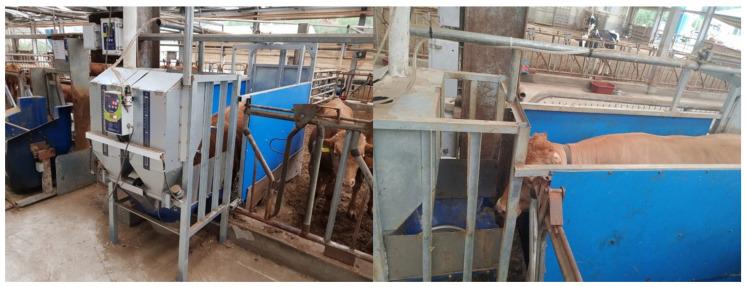
Automatic concentrate feeding system (Dawoon Co., Incheon, Republic of Korea) used in this experiment.

The external dimensions of the ACFS (Dawoon Co., Incheon, Republic of Korea) used in this study were 0.8 m in width, 2 m in length, and 1.9 m in height (Figure 1). By default, the gate of the ACFS remains open when animals are absent. However, once an RFID neck tag is detected by the feeder for 2 s, the system recognizes the presence of the steer and closes the gate to ensure that only one steer accesses the feeder at a time. The gate reopens if the designated amount of concentrate mix is entirely consumed or if the RFID neck tag is not sensed for more than 70 s, regardless of the presence of the steer inside the station. The feeder dispenses a preset amount of concentrate mix to each steer based on its daily allowance and the number of dividing intervals. During an interval, the feeder allocates the mix to multiple fixed-size portions (80–90 g) until reaching the steer’s allocated portion for that interval. For example, when a steer was allowed to consume 2 kg in a single interval and the fixed portion size was 80 g, the feeder dispensed the mix 25 times within that interval. The duration of each interval was calculated by dividing 24 h by the number of intervals, and the daily feed allowance was uniformly distributed over these periods. Any unconsumed portion of an interval can be consumed in the subsequent interval. The ACFS automatically logs the steer’s entry time, duration of stay, and amount of concentrate mix provided during each visit. However, a steer’s entry and stay times are typically recorded only when it receives a feed allocation. If a steer exhausts its set feed amount for an interval, the system logs its entry time and marks its stay duration as 10 min. Subsequent entries by the same steer within 10 min were discarded, and the data remained unrecorded.

### 2.3. Data Collection and Processing

This experiment aimed to assess the impact of two interval settings (i.e., four and six intervals) of the ACFS on concentrate mix using a switchback design [17]. Each period lasted seven days, including four days for adaptation and three days for data collection. The four-day adaptation period was chosen based on a preliminary study, which indicated that the feed intake pattern (i.e., time of day, frequency, and duration of daily feed station visits) stabilized within three days after changing the number of dividing intervals of the ACFS. In this study, although two types of concentrate mix with different starch contents were provided, feed treatment did not significantly influence feed intake and feeding behavior (*p* > 0.05; data not shown). Therefore, we integrated and analyzed the data across different feed treatments.

**Figure 2:**
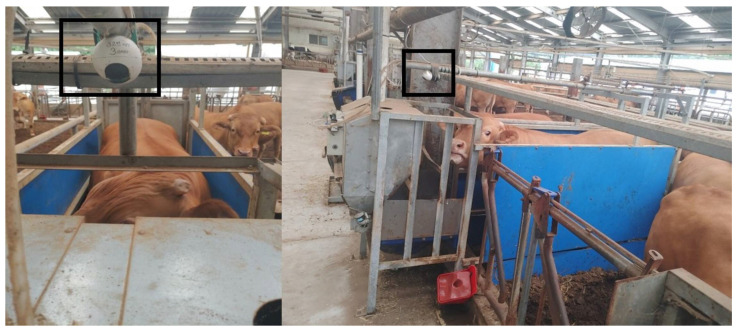
Location of the installed video camera (MJSXJ01CM, Xiaomi Technology Co. Ltd., Beijing, China) in automatic concentrate feeding system (Dawoon Co., Incheon, Republic of Korea). The black frame represents the video camera used in this experiment.

Visual observation was employed to determine the amount of feed consumed by each animal. Because the ACFS used in this study only recorded the amount of concentrate mix given to each animal at each visit, it was unknown whether any concentrate mix remained after each visit. To address this, we installed a video camera (MJSXJ01CM, Xiaomi Technology Co. Ltd., Beijing, China) on each ACFS, which continuously recorded the amount of concentrate mix remaining in the feed bowl (Figure 2). The feed bowl was marked to denote the residual amount of the concentrate mix, which ranged from 0.2 to 4 kg. Three trained observers independently and manually examined the video recordings during the data collection period to measure the residual amount of concentrate mix after each steer visit. The actual intake at each visit was determined by calculating the difference between the automatically recorded amount of feed offered and the manually determined amount of feed residue. One animal was excluded from the data analysis because of a health problem. To determine the accuracy of the residual amount of the concentrate mix, the Pearson correlation between the amounts of concentrate mix residue determined by each independent observer for each event was analyzed. Because the correlation was significantly high (r > 0.94; Table 3), the residual amount of the concentrate mix for each sample was calculated by averaging the residuals determined by the three observers.

### 2.4. Feeding Behavior Analysis

The duration of steers in the ACFS, defined as the time spent per day regardless of feed consumption, was manually calculated by observers analyzing the footage recorded through video cameras. The visit frequency to the ACFS was defined as the total number of times a steer visited the ACFS in one day, irrespective of whether the steer consumed concentrate mix in the ACFS (i.e., both instances of consuming and not consuming feed were counted). However, if the time interval between the exit time of the preceding visit and the entry time of the subsequent visit was less than 5 min, the two visit records were consolidated and considered as one visit [18]. This data processing was conducted using basic data mining functions (strip, split, etc.) in Python (ver. 3.8).

The meal frequency of forage was defined as the number of times the steer consumed feed from the FIMS during the day. Unlike the visit frequency in the concentrate mix feeding behavior, the meal frequency in the forage feeding counted only instances in which steers visited the FIMS and consumed forage (i.e., it was not counted if the forage was not consumed). Additionally, for forage behavior, even if the time interval between the preceding and subsequent visits was less than 5 min, we did not integrate the two visit records but calculated them as two separate visits as recorded. The meal frequency of forage was analyzed using basic data mining functions (strip, split, etc.) in Python (ver. 3.8) based on feeding behavior data recorded using the FIMS.

### 2.5. Statistical Analysis

The data were analyzed using PROC MIXED in SAS (SAS Institute Inc., Cary, NC, USA). The linear model was as follows:

*y*_ij_ = *μ* + *τ*_i_ + *e*_ij_

where *y*_ij_ is the jth observation (j = 1–29) in the ith treatment (i = 1–2), *μ* is the overall mean, *τ*_i_ is the fixed effect of the ith treatment, and *e*_ij_ is the unexplained random effect on the jth observation in the ith treatment.

Individual means were compared using the Tukey’s test. Statistical significance was declared at *p* < 0.05, and a trend was declared at 0.05 ≤ *p* < 0.1.

## 3. Results and Discussion

### 3.1. Intake and Feeding Behavior with Concentrate Mix

In this study, the actual intake of the animals in the ACFS was determined by three trained observers who ascertained the residual concentrate mix for each animal using recorded videos. The Pearson correlation coefficients among the residuals of the concentrate mix determined by the three trained observers for each animal ranged from 0.94 to 0.97 (Table 3). These results indicate a consistency in the determination of the residual concentrate mix and, consequently, in the observed concentrate mix intake for each animal during the data collection period.

The interval setting of the ACFS did not have a significant effect on the observed and recorded concentrate mix dry matter intake (DMI) of the steers (*p* > 0.05; Table 4). However, increasing the number of intervals in the ACFS significantly reduced the residuals of concentrate mix (0.46 vs. 0.27 kg/d, respectively; *p* = 0.014; Table 4). These results indicate that the absence of a difference between the observed and recorded average concentrate intake in this study can be attributed to some individuals not consuming all of the provided concentrate mix and others consuming the residue left by fellow animals. Historically, large-scale herd-based management was predominant; however, in modern practices, individual-based management has become crucial [19]. Consequently, the adoption of ACFSs, which allow for the setting and feeding of individual feed amounts, is increasing in the cattle farming industry [13]. Nevertheless, the limitations of ACFSs in accurately measuring individual intake pose challenges to precision feeding for individual animal management. Overcoming these challenges is crucial when adopting an ACFS for individual animal management. The results of this study suggest that increasing the interval setting of an ACFS could be a viable approach to ensure animals consume close to the targeted feed intake. However, even when the concentrate mix was divided and fed six times a day by the ACFS, some individuals still left residuals or consumed the remaining amounts left by other individuals; therefore, further studies should be conducted to explore ways to completely resolve this issue.

In this study, the daily allowance of concentrate mix was set at 9.2 kg DM/d. However, regardless of the ACFS interval setting, both the recorded and actual concentrate mix intakes were approximately 8.8 kg DM/d, representing 96% of the target quantity. These results differ from previous research findings in dairy cows, whereby the cows consumed approximately 55–85% of the allowed daily concentrate mix with an ACFS [9,11,12]. The discrepancy in the research findings between dairy and beef cattle could be attributed to milking requirements. Dairy cows need to be milked to receive a concentrate mix supply from an ACFS integrated into an AMS. In contrast, beef cattle do not require such conditions to receive a concentrate mix from an ACFS. Variations in these conditions may contribute to a reluctance in dairy cows to consume the concentrate mix. The results of the concentrate mix feeding behavior in our study support these findings. Both the duration in the ACFS (72.61 vs. 80.91 min/d, respectively) and the visit frequency to the ACFS (6.30 vs. 8.46 events/d, respectively) significantly increased as the number of intervals for the ACFS increased (*p* < 0.01; Table 4). These results indicate that beef cattle visited the ACFS more frequently than the set number of intervals and that increasing the number of intervals resulted in a corresponding increase in visit frequency and duration. Unlike the current study, previous studies [9,11,12] with dairy cows indicated a strong motivation for cows to consume concentrate mix when the amount of daily concentrate mix increased without changing the interval setting for the ACFS. However, this did not result in an increase in voluntary visits to an AMS by cows. These findings indicate that, despite a potentially stronger motivation for cows to consume concentrate mix in an ACFS compared to the motivation to empty the udder [20], milking can potentially interfere with the cows’ willingness to visit an AMS. This, ultimately, led to a reduced achievement rate of actual intake compared to the target intake in the ACFS. Furthermore, dairy cows require a long period between the previous milking and the next milking to consume the concentrate mix supplied by the ACFS [21]. These systems may result in a larger discrepancy between the actual and target intakes in ACFSs for cows that do not voluntarily milk periodically throughout the day or have an insufficient milk yield. However, the ACFS used in this study allowed the animals to consume the concentrate mix from the previous interval in the next interval, even if they did not consume the concentrate mix supplied in the previous interval. The differences in these systems can make it more challenging for dairy cows to reach the target intake in ACFS compared to beef cattle.

Another factor contributing to the observed intake differences between dairy and beef cattle may arise from their primary diets designed to meet nutritional requirements. Generally, dairy cows that are milked utilizing an AMS primarily meet their nutritional requirements through a partial mixed ration (PMR); the concentrate mix is used as an adjunct to voluntary attendance at an AMS and to meet nutritional requirements that are not met by PMR [22]. On the other hand, during the fattening period, beef cattle fed separate diets of forage and concentrate mix, rather than a TMR, cannot meet their nutritional requirements through forage. Consequently, they mainly meet their nutritional requirements through the concentrate mix [23]. Although cattle have a high preference for a concentrate mix, differences in the feed types that provide these main energy sources can lead to variations in feed intake. Hare et al. [24] reported that compared to dairy cows fed PMR with a high energy content, those fed PMR with a low energy content consumed more concentrate mix through an AMS and less PMR. The results of the present study suggest that these differences may apply to feeding via the ACFS in beef cattle.

### 3.2. Intake and Feeding Behavior with Forage and Total Intake

As the number of intervals set for the ACFS increased, the forage DMI (1.48 vs. 1.21 kg/d, respectively; *p* = 0.001) and the frequency of visits to the FIMS (15.12 vs. 13.92 events/d, respectively; *p* = 0.004; Table 4) significantly decreased. The significant difference in forage DMI based on the interval setting of the ACFS resulted in a significant decrease in total DMI (10.26 vs. 9.97 kg/d, respectively; *p* = 0.018; Table 4), despite no significant difference in mean recorded and observed concentrate DMI. Increasing the daily feed amount of concentrate mix using an ACFS in an AMS leads to an increase in the intake of concentrate mix by animals, resulting in a reduction in PMR intake [22,24,25]. However, in this study, the daily concentrate mix supply in the ACFS remained constant regardless of the interval setting. Thus, the decrease in forage DMI during the six intervals cannot be attributed to changes in the concentrate mix supply. Nevertheless, the decrease in forage DMI when fed six times may be attributed to steers visiting the ACFS more frequently than when fed four times, leading to more frequent consumption of the concentrate mix. Generally, because cattle prefer concentrate mix over forage when the forage lacks sufficient nutritional content [26], they tend to prioritize the consumption of concentrate mix over forage. Therefore, compared to the four time intervals, when supplying concentrate mix more frequently with the six time intervals, steers are likely to prioritize the consumption of compound feed over forage to alleviate hunger. This more frequent consumption of concentrate mix can shorten the length of time of feeding hunger and, thus, result in lower motivation to consume forage. In this study, an increase in the interval setting for the ACFS group resulted in a significant increase in the frequency and duration of ACFS visits (*p* < 0.05; Table 4). These results indicate that fattening Hanwoo steers effectively adapted to the increased intervals in the ACFS, consuming concentrate mix more frequently with the six time intervals, depending on the interval setting of the ACFS. Consequently, although the supply of concentrate mix per feeding session over the six time intervals was lower than that of the four time intervals, the amount of time between the two meals when steers were fed concentrate mix was shortened, which may have shortened the fasting period and led to a decrease in forage intake.

## 4. Conclusions

Regardless of the interval setting for an ACFS, the actual concentrate mix DMI of the fattening Hanwoo steers was 96% of the target intake. The increase in the number of intervals in the ACFS did not affect the concentrate mix DMI but increased the visit frequency to and duration in the ACFS. Additionally, the visit frequency to the forage feed bunk and DMI of the forage, as well as the total DMI, decreased as the number of intervals increased for the ACFS. Even with this increase in intervals for the ACFS, residue of the concentrate mix was still present; however, the residue significantly decreased with the interval setting of six compared with the interval setting of four. Further research is necessary to explore alternative methods that can comprehensively address this issue for the successful implementation of ACFSs for individual management in beef cattle farming.

## Figures and Tables

**Table 1:** Compositions (g/kg dry matter or as stated) of the experimental diets.

Items ^1^	Concentrate Mix	
	Commercial	High Starch
Corn, flaked	290	292
Corn, ground	30	114
Wheat, ground	60	69
Barley, flaked	41	41
Lupin, flaked	21	21
Wheat flour	41	13
Corn gluten feed	207	147
Palm kernel meal	103	93
Rapeseed meal	11	41
Corn germ meal	30	0
DDGS	0	20
Cottonseed, whole	30	30
Rice bran	30	30
Mixed hull	32	14
Limestone	30	31
Molasses	18	18
CMS	11	11
CSL	3	3
Salt	7	8
Sodium bicarbonate	3	3
Vitamin and mineral mix ^‡^	3	3

^1^ DDGS, distillers dried grains; CMS, condensed molasses solubles; CSL, corn steep liquor. ^‡^ 33,330,000 IU/kg vitamin A; 40,000,000 IU/kg vitamin D; 20.86 IU/kg vitamin E; 20 mg/kg Cu; 90 mg/kg Mn; 100 mg/kg Zn; 250 mg/kg Fe; 0.4 mg/kg I; 0.4 mg/kg Se.

**Table 2:** Analyzed chemical compositions (g/kg DM or as stated) of the experimental diets.

Items ^1^	Concentrate Mix		Forage
	Commercial	High Starch	
DM, g/kg as fed	888	883	900
OM	919	918	936
CP	150	154	56
SOLP	53	53	20
NDICP	22	20	15
ADICP	12	12	12
aNDF	283	273	739
ADF	155	138	517
ADL	45	40	82
Ether extract	44	47	8
Ash	81	82	64
Ca	13	14	5
*p*	6	6	1
K	10	9	8
Na	4	5	4
Cl	7	8	3
S	3	3	2
Mg	3	3	1
TDN	715	728	489
NEm, MJ/kg DM	7.3	7.5	3.9
NEg, MJ/kg DM	4.7	4.9	1.6
Total carbohydrates	725	717	872
NFC	464	464	147
Carbohydrate fraction, g/kg carbohydrate			
CA	64	62	42
CB1	509	540	11
CB2	67	45	116
CB3	212	220	604
CC	148	133	226
Protein fraction, g/kg CP			
PA + B1	352	348	359
PB2	503	522	369
PB3	61	51	52
PC	83	79	221

^1^ DM, dry matter; OM, organic matter; CP, crude protein; SOLP, soluble CP; NDICP, neutral detergent insoluble CP; ADICP, acid detergent insoluble CP; aNDF, neutral detergent fiber analyzed using a heat stable amylase and expressed inclusive of residual ash; ADF, acid detergent fiber; ADL, acid detergent lignin; TDN, total digestible nutrients; NEm, net energy for maintenance; NEg, net energy for growth; NFC, non-fiber carbohydrate; CA, carbohydrate A fraction. Ethanol soluble carbohydrates—CB1, carbohydrate B1 fraction; starch—CB2, carbohydrate B2 fraction; soluble fiber—CB3, carbohydrate B3 fraction; available insoluble fiber—CC, carbohydrate C fraction; unavailable carbohydrate—PA+B1, protein A and B1 fractions; soluble CP—PB2: protein B2 fraction; intermediate degradable CP—PB3, protein B3 fraction; slowly degradable fiber-bound CP—PC, protein C fraction; unavailable CP.

**Table 3:** Pearson correlation between residuals of concentrate mix recorded by three trained observers.

	Observer 1	Observer 2	Observer 3
Observer 1	1.00		
Observer 2	0.94	1.00	
Observer 3	0.97	0.96	1.00

**Table 4:** Effect of meal intervals of concentrate mix feeding per day by automatic feeding system on feed intake, residual concentrate mix, and bunk visit frequency for forage.

Items ^1^	Number of Meal Intervals of Concentrate Mix Per Day		SEM ^2^	*p*-Value
	Four	Six		
Total dry matter intake, kg/day	10.26	9.97	0.118	0.018
Concentrate				
Recorded intake, kg DM/day	8.79	8.77	0.123	0.864
Observed intake, kg DM/day	8.78	8.76	0.128	0.884
Residual concentrate, kg/day	0.46	0.27	0.077	0.014
Visit frequency to ACFS, events/day	6.30	8.46	0.397	<0.001
Duration of animals in the ACFS, min/day	72.61	80.91	2.536	0.002
Forage				
Intake, kg DM/day	1.48	1.21	0.078	0.001
Meal frequency of forage, events/day	15.12	13.92	0.423	0.004

^1^ DM, dry matter; ACFS, automatic concentrate feeding system. ^2^ SEM, standard error of the mean.

## Data Availability

The data presented in this study are available on request from the corresponding author.
